# Parietal Cortex Connectivity as a Marker of Shift in Spatial Attention Following Continuous Theta Burst Stimulation

**DOI:** 10.3389/fnhum.2021.718662

**Published:** 2021-09-08

**Authors:** Jessica Mariner, Tobias Loetscher, Brenton Hordacre

**Affiliations:** ^1^Innovation, IMPlementation And Clinical Translation in Health (IIMPACT in Health), Allied Health and Human Performance, University of South Australia, Adelaide, SA, Australia; ^2^Behavior-Brain-Body Research Center, Justice and Society, University of South Australia, Adelaide, SA, Australia

**Keywords:** non-invasive brain stimulation, spatial attention, functional connectivity, neuroplasticity, electroencephalography

## Abstract

Non-invasive brain stimulation is a useful tool to probe brain function and provide therapeutic treatments in disease. When applied to the right posterior parietal cortex (PPC) of healthy participants, it is possible to temporarily shift spatial attention and mimic symptoms of spatial neglect. However, the field of brain stimulation is plagued by issues of high response variability. The aim of this study was to investigate baseline functional connectivity as a predictor of response to an inhibitory brain stimulation paradigm applied to the right PPC. In fourteen healthy adults (9 female, aged 24.8 ± 4.0 years) we applied continuous theta burst stimulation (cTBS) to suppress activity in the right PPC. Resting state functional connectivity was quantified by recording electroencephalography and assessing phase consistency. Spatial attention was assessed before and after cTBS with the Landmark Task. Finally, known determinants of response to brain stimulation were controlled for to enable robust investigation of the influence of resting state connectivity on cTBS response. We observed significant inter-individual variability in the behavioral response to cTBS with 53.8% of participants demonstrating the expected rightward shift in spatial attention. Baseline high beta connectivity between the right PPC, dorsomedial pre-motor region and left temporal-parietal region was strongly associated with cTBS response (*R*^2^ = 0.51). Regression analysis combining known cTBS determinants (age, sex, motor threshold, physical activity, stress) found connectivity between the right PPC and left temporal-parietal region was the only significant variable (*p* = 0.011). These results suggest baseline resting state functional connectivity is a strong predictor of a shift in spatial attention following cTBS. Findings from this study help further understand the mechanism by which cTBS modifies cortical function and could be used to improve the reliability of brain stimulation protocols.

## Introduction

Transcranial magnetic stimulation (TMS) provides the ability to interact with neural tissue through the intact scalp (Rothwell et al., 1991). Repetitive and patterned forms of TMS, such as theta burst stimulation, are thought to be capable of inducing behavioral and physiological aftereffects *via* mechanisms that resemble synaptic plasticity. In a seminal study, intermittent theta burst stimulation was found to increase corticospinal excitability, while continuous theta burst stimulation (cTBS) decreased corticospinal excitability (Huang et al., 2005). Modulation of excitability by theta burst stimulation appears to be short-lasting (∼30–60 min), with aftereffects blocked by administration of the NMDA receptor antagonist Memantine (Huang et al., 2007). Dependency on the NMDA receptor suggests theta burst stimulation aftereffects likely involve changes at synaptic connections in the cortex. Given the relatively short time course of stimulation aftereffects, it appears that the neuroplastic response involves mechanisms similar to those responsible for early-phase long-term potentiation and long-term depression (Goldsworthy et al., 2014b).

The ability to induce neuroplasticity within the human cortex provides an opportunity to investigate behavioral roles of different brain regions or deliver therapeutic treatment. For example, cTBS to suppress cortical activity of the right posterior parietal cortex (PPC) interrupts attentional processes, affecting visuospatial attention and inducing transient spatial neglect-like symptoms in healthy adults (Fierro et al., 2000; Nyffeler et al., 2008; Cazzoli et al., 2009a; Chechlacz et al., 2015). Along similar lines, theta burst stimulation has been used to treat visuospatial neglect symptoms in people with stroke, with a recent systematic review reporting that most studies delivered cTBS to suppress hyper-activity of the contralesional PPC (Cotoi et al., 2019). Along with improvements in spatial neglect, there is also evidence that this treatment can increase participation in activities of daily living (Cazzoli et al., 2012). These promising findings provide hope for using theta burst stimulation as a tool to probe the human brain or provide therapeutic treatments.

However, recent evidence indicates there is significant variability in physiological and behavioral responses to theta burst stimulation and non-invasive brain stimulation in general (López-Alonso et al., 2014; Hordacre et al., 2015; Lopez-Alonso et al., 2015). For example, several studies evaluating modulation of motor evoked potentials (MEPs) following theta burst stimulation report that approximately half of the tested participants respond as expected (Hamada et al., 2013; Hinder et al., 2014; Hordacre et al., 2017a, 2021a; Jannati et al., 2017). Similarly, many studies fail to demonstrate behavioral change at a group level, possibly due to variability in how individuals response to stimulation (Benninger et al., 2011; Talelli et al., 2012; Bologna et al., 2015, 2016). Much work has been performed to understand this response variability in order to gain further understanding of how non-invasive brain stimulation influences synaptic connections in the brain and subsequently help develop more reliable stimulation protocols. In 2010, a review outlined that determinants of response to brain stimulation included age, gender, history of synaptic activity, aerobic exercise, pharmacology and genetics (Ridding and Ziemann, 2010). Since this time, additional factors such as the inter-individual differences in the cortical network activated by brain stimulation (Hamada et al., 2013; Hordacre et al., 2017a), neural variability (Hordacre et al., 2017a) and functional connectivity of the stimulated network (Cárdenas-Morales et al., 2014; Nettekoven et al., 2015; Hordacre et al., 2021a) have also been reported to influence theta burst stimulation response.

Although much of this work has investigated determinants of the physiological response to theta burst stimulation applied to the motor cortex, it may prove informative for investigation of brain stimulation applied to networks outside the motor system. Similar issues of physiological and behavioral response variability have been reported following stimulation of other brain regions such as the PPC (Cazzoli et al., 2009b; Rizk et al., 2013; Killington et al., 2016; Nyffeler et al., 2019; Vatanparasti et al., 2019). Of note, there is preliminary indication that the behavioral response to PPC stimulation might be associated with pre-stimulus functional connectivity (Rizk et al., 2013). Specifically, it was observed that two of nine participants who did not respond to right PPC stimulation as expected had lower baseline alpha band connectivity of the right temporoparietal junction. This finding is worthy of further investigation, particularly as spatial deficits have been suggested to reflect interhemispheric network imbalances (Corbetta and Shulman, 2011). Furthermore, it may be worth exploring how interhemispheric network connectivity might interact with other, already known, determinants of response variability.

This study aims to investigate functional connectivity as a marker of change in spatial attention following cTBS. Resting state functional connectivity was assessed using electroencephalography which provides high temporal resolution, enabling the investigation of specific frequency bands. We have previously demonstrated that resting state functional connectivity of the stimulated motor network in alpha and high beta bands was a strong predictor of response to anodal tDCS in healthy adults and people with stroke (Hordacre et al., 2017b, 2018). Although performed in the motor network, these findings provide some rationale to explore the role of functional connectivity as a predictor of response for brain stimulation to other neural target. In support, preliminary evidence suggests alpha band connectivity as a possible marker of response to PPC stimulation (Rizk et al., 2013). In line with these findings, we hypothesized stronger baseline alpha and high beta band resting state functional connectivity with a seed overlying the stimulated PPC would be a marker of change in spatial attention following cTBS. Identifying determinants of response to theta burst stimulation of the PPC is an important step toward gaining insight into how this potential neuromodulation treatment interacts with the human cortex.

## Materials and Methods

### Participants

Healthy adult participants were eligible for inclusion if they were aged 18–30 years, right-handed (self-reported), not taking neuroactive medications, not under the influence of alcohol or excessive caffeine intake, did not have any neurological or psychiatric conditions and were deemed safe for brain stimulation (Rossi et al., 2011). We restricted participant age, medications, alcohol and caffeine consumption given evidence that these impair neuroplasticity or alter corticospinal excitability (Ziemann, 2004; Specterman et al., 2005; Lücke et al., 2014; Jannati et al., 2019). Informed consent was obtained in accordance with the World Medical Association Declaration of Helsinki, and ethics approval was provided by the University of South Australia’s Human Research Ethics Committee (approval date 9/10/2018, ID 201539). This study was prospectively registered on the Australian, New Zealand Clinical Trials Registry (ACTRN12619000661178).

### Procedures

**FIGURE 1:**
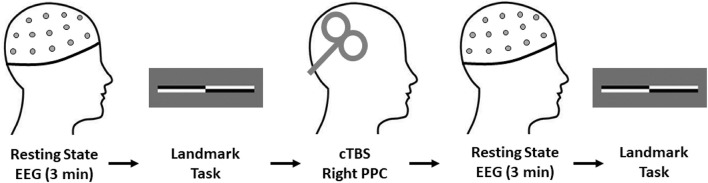
Experimental procedures. All participants completed the experimental procedures in the order shown. Total experimental duration was ∼60 min. Note, the EEG cap remained fixed on the participants head for the duration of the experimental procedures to ensure consistent electrode placement throughout data collection. cTBS, continuous theta burst stimulation; EEG, electroencephalography.

To control for known determinants of cTBS response, all experimental sessions were conducted between 11 am and 3 pm as diurnal variation in endogenous cortisol levels and circadian rhythms are known to affect neuroplasticity responses (Sale et al., 2008; Clow et al., 2014). Furthermore, stress and physical activity levels were assessed with the Perceived Stress Scale Questionnaire (PSSQ) (Cohen et al., 1983) and an International Physical Activity Questionnaire (IPAQ) (Craig et al., 2003). Following this, resting state EEG was recorded continuously for 3 min, followed by the Landmark Task (LMT) as a behavioral assessment of spatial attention. cTBS was then delivered to the right PPC, with a post-cTBS EEG block recorded for 3 min followed by participants performing the LMT (see Figure 1). All post-intervention measures were completed within 30 min of cTBS, as this is within the window of lasting behavioral effects after TBS (Ridding and Rothwell, 2007).

### Electromyography

Surface electromyography (EMG) was used to record MEPs from the participant’s first dorsal interosseous (FDI) muscle of the left hand to determine resting motor threshold (RMT) of the right motor cortex. The skin over the FDI was prepared with alcohol wipes and NuPrep paste. A ground strap was fixed around the left wrist, with two Ag-AgCl surface electrodes (Ambu, Ballerup, Denmark) placed on the muscle belly and tendon of the left FDI. Signals were sampled at 5 kHz (CED 1401; Cambridge Electronic Design, Cambridge, United Kingdom), amplified (x 1,000) (CED 1902; Cambridge Electronic Design or Digitimer D360, Welwyn Garden City, Herts, United Kingdom) and filtered (20–1,000 Hz).

### Continuous Theta Burst Stimulation

cTBS was delivered to the right PPC using a bi-phasic wave form delivered by a Magstim super rapid stimulator (Magstim Company, Dyfed, United Kingdom) connected to an air-cooled, figure-of-eight coil. First, RMT of the right motor cortex was determined by positioning the coil perpendicular to the scalp with the handle pointing 45 degrees postero-laterally (Brasil-Neto et al., 1992). Pulses were delivered at 0.2 Hz ± 10% to evoke responses in the relaxed FDI. To find the optimal position for evoking MEPs, the coil was moved in small increments anterior-posterior and medial-lateral. Once the optimal position was determined, coil position was marked and held in place. RMT was defined as the minimum intensity that evoked a MEP with peak-to-peak amplitude ≥50 μV in at least 5/10 consecutive trails. cTBS was then delivered to the right PPC by positioning the coil at P4 according to the 10/20 EEG system. Stimulation was delivered at 70% RMT for a duration of 40 s. Stimulation intensity of 70% RMT was selected as it is approximately similar to the absolute intensity when determining 80% active motor threshold, but avoids muscle activation (to obtain active motor threshold) which could abolish aftereffects of cTBS and lead to less consistent responses (Goldsworthy et al., 2012, 2014a; Fried et al., 2019). A total of 600 pulses were applied in bursts of three pulses at 50 Hz, repeated at 5 Hz (Huang et al., 2005).

### Electroencephalography

Brain activity was recorded using an ASA-lab EEG system (ANT Neuro, Enschede, Netherlands), with 64 sintered Ag-AgCl electrodes in standard 10/10 positions. EEG conductive gel was applied to keep signal impedance kept below 5 kΩ throughout data collection. Signals were sampled at 2,048 Hz, amplified (x20) filtered (online filtered 1–45 Hz) and online referenced to CPz. Continuous data was recorded for 3 min. During data collection, participants were seated in a comfortable chair, asked to relax, keep their eyes open and looking at a fixation cross straight ahead, limit talking and try not to engage in any mental activities. Recorded EEG data was stored on a computer for offline analysis.

### Electroencephalography Processing and Analysis

EEG data was exported to MATLAB 8.1.0 (MathWorks, Inc., Natick, MA, United States) for pre-processing and analysis. First, unused channels (HEOG, VEOG, M1, and M2) were removed, followed by visual inspection of the data and the removal of noisy channels. Data were then filtered 1–45 Hz using the EEGLAB pop_eegfiltnew function (Delorme and Makeig, 2004), segmented into 180 one second epochs and submitted to an independent component analysis using the EEGLAB fast ICA function (Delorme and Makeig, 2004) where non-physiological data such as eye blinks and muscle contractions were removed. Bad channels were then re-interpolated. The de-biased weighted phase lag index (dwPLI) was calculated between electrodes as a conservative estimate of functional connectivity (Vinck et al., 2011). The dwPLI was calculated using the FieldTrip toolbox of MATLAB (Oostenveld et al., 2011) and is based on phase consistency. It biases against zero phase lag relationships to limit the effects of volume conduction and common reference problems. dwPLI values range from 0 (no phase coupling) to 1 (maximum phase coupling) and were calculated for the frequency bands of delta (1–3 Hz), theta (4–7 Hz), alpha (8–13 Hz), low beta (14–20 Hz), high beta (21–30 Hz) and gamma (31–45 Hz).

### Behavioral Data

The LMT was used to measure spatial attention (Harvey et al., 1995; Mccourt and Jewell, 1999). During testing, the participant sat on a standard chair with a height adjustable lever, in front of a computer screen that was aligned with their mid-sagittal plane at eye level, 500 mm away. The participant’s head position was maintained using an adjustable chinrest, so that the center of the screen was in line with their mid-sagittal line. The computer monitor and keyboard were in front of the participant, parallel with their mid-sagittal plane. The stimuli were pre-bisected lines that were based on a study by Nicholls et al. (2014). The lines were made up of diagonally opposing white and black segments, with a total length of 180 mm and height of 5 mm. They were presented against a gray background. E-prime 2.0 software (Psychology Software Tools, Sharpsburg, MD, United States) was used to control the presentation of the pre-bisected line stimulus on an LCD screen of diagonal width 545 mm. A pre-bisected line stimulus was presented on the vertical screen center for 2,000 ms, followed by a blank gray screen. The participant then needed to indicate if they perceived the left or the right line segment as longer, by pressing the “F” or “J” keys, respectively, with their index fingers. Responses made after 2,000 ms were discarded and a new, identical trial replaced it. The participant was then reminded to make a faster response in future trials.

Each participant performed 144 trials before and after cTBS. These trials comprised of four repetitions of the 36 unique factorial combinations of three bisection deviations (1, 2 or 3 mm from true center), two sides (left or right side longer), two polarities (black or white on top left line segment) and three horizontal stimuli screen locations (−2, 0, +2 mm to the left or right of the center). The line was never bisected in the true center, as it was always shifted slightly to one side. The horizontal stimuli location was to prevent the participant from using a landmark on the screen as a cue (Nicholls et al., 2014). There were 72 practice trials performed after the baseline EEG recording, but before the start of the first LMT session.

To measure whether a participant had a spatial attentional bias toward the left or right side, a response bias was calculated as 100 × (number of right responses minus the number of left responses) divided by the total number of trials (Nicholls et al., 2014). These values can range from −100 to +100. Negative values indicate a spatial bias toward the left, whereas positive values indicate a rightward spatial bias. The pre-cTBS scores were subtracted from the post-cTBS scores (ΔLMT). A positive ΔLMT was indicative of a rightward shift in spatial attention, the anticipated response following cTBS. To control for participants that may have misunderstood instructions for the task, a threshold of 30% correct responses (i.e., significantly less than chance level) was used. If a participant was unable to meet this criterion, they were removed from the analysis.

### Data Analysis

#### Partial Least Squares Regression Analysis

A partial least squares regression analysis (PLS) was conducted using the N-way Toolbox for MATLAB (Andersson and Bro, 2000). PLS regression identified a model of connectivity between P4 (seed electrode, cTBS stimulation site) and all others, to maximally predict variance in ΔLMT. PLS analysis has several advantages that make is particularly suitable for the current analysis. These advantages are: (1) ability to handle a greater number of independent variables than observations without increased risk of Type I error, and (2) capacity to handle non-orthogonal independent variables (Cramer, 1993). Similar to previous work, a conservative threshold of 0.7 for PLS analysis was determined relative to the maximal correlation co-efficient (Hordacre et al., 2017b, 2018). Separate PLS regression analyses were performed for each frequency band of interest. Data was mean centered and entered into a direct orthogonal signal correction (Westerhuis et al., 2001) to achieve more efficient PLS modeling. The first component was used for each PLS model. For each identified PLS model, cross validation was performed using a leave-one-out and predict analysis to provide an indication of predictive capacity of the model. To demonstrate robustness of PLS models, the threshold range capable of producing the same electrode clusters was identified. Finally, for each PLS model, clusters of electrodes defined as having at least two adjacent electrodes in space were identified. The mean dwPLI for each cluster was then correlated against the dependent variable.

#### Statistical Analysis

Statistical analysis was undertaken using SPSS software (IBM Corp., 2016, IBM SPSS Statistics for Windows, Version 24.0, Armonk, NY, United States) and MATLAB 8.1.0 (MathWorks, Inc., Natick, MA, United States). The statistical significance level was set to *p* < 0.05, and the Shapiro–Wilk test was used to check the normality of the data. A paired *t*-test was conducted to compare pre-intervention and post-intervention LMT results. Pearson correlations were performed to determine the association between ΔLMT and age, RMT, PSSQ scores and IPAQ scores. The effect of sex on ΔLMT was investigated with an independent *t*-test. A multiple regression was conducted to further explore all known determinants of cTBS response that were documented in this study. The dependent variable was ΔLMT and independent variables were sex, age, RMT, PSSQ score, IPAQ score, baseline dwPLI of the identified electrode clusters. To further understand the role of an identified dwPLI network, we conducted an exploratory analysis to investigate change in dwPLI of the identified network (paired *t*-test) at the group level and by analyzing responders and non-responders separately. We also conducted a Pearson correlation to determine the association between change in dwPLI and ΔLMT. Where appropriate, Bonferroni corrections were applied to correct for multiple comparisons.

## Results

Fourteen healthy adults participated in this study (9 females, aged 24.8 ± 4.0 years). One participant (P09) was excluded from the analyses, due to performing below chance level with an accuracy of 16% at baseline, possibly from a misunderstanding of instructions (see Supplementary Table 1). For the remaining participants, the average biphasic RMT of the right motor cortex was 57.2% (SD 10.8) maximum stimulation output. The mean PSSQ was 16.8 ± 3.8 (range of 10–21), indicating moderate levels of perceived stress at a group level. The mean IPAQ score was 4,423.08 ± 3633.7 MET-min/week. Individual participant details are provided in Supplementary Table 1. No participants reported any adverse effects from the experiment session.

### Behavioral Response to cTBS

**FIGURE 2:**
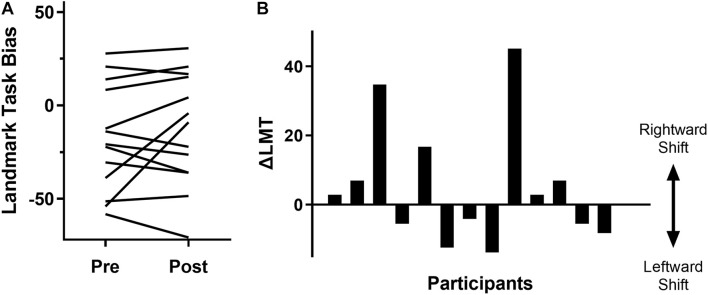
Variability of behavioral response to cTBS as measured by the LMT. **(A)** Pre and post cTBS LMT bias results for each participant (each line represents a participant). An increase represents a rightward bias shift and a decrease represents a leftward bias shift. **(B)** Change in LMT bias (post-pre intervention) for each participant, displaying a rightward or leftward shift in attention.

As anticipated, at a group level there was a non-significant change in LMT, with a pre-cTBS LMT of −17.8 ± 28.9, a post-cTBS LMT of −12.8 ± 30.4 and a ΔLMT of 5.1 ± 17.8 [*t*_(12)_ = −1.03, *p* = 0.32]. Further investigation revealed substantial inter-individual variability in the behavioral data (see Figure 2). Specifically, seven participants (53.8%) demonstrated the expected rightward shift (mean ΔLMT = 16.6 ± 16.9). Six participants (46.2%) did not respond as expected, with a mean leftward shift of ΔLMT = −8.4 ± 4.0. There were no associations between ΔLMT and age (*r* = 0.09, *p* = 0.76), sex [*t*_(11)_ = 1.11, *p* = 0.29], RMT (*r* = 0.15, *p* = 0.62), PSSQ scores (*r* = 0.19, *p* = 0.54) or IPAQ score (*r* = 0.04, *p* = 0.89).

### EEG Connectivity as a Predictor of cTBS Response

**FIGURE 3:**
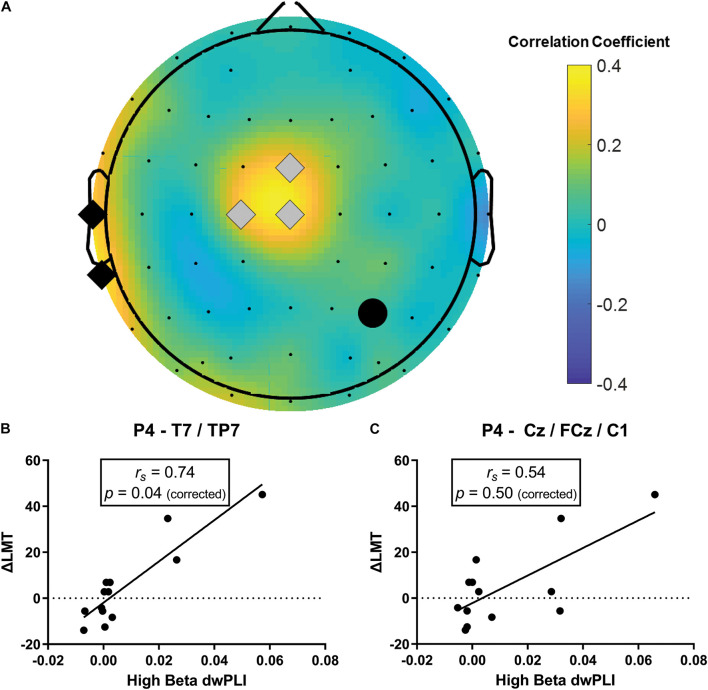
Baseline high beta dwPLI was associated with the behavioral response to cTBS (ΔLMT). **(A)** Topoplot of PLS connectivity model that maximally accounted for variance in ΔLMT. Note, the black circle represents the seed electrode (P4, site of cTBS application), gray diamonds represent the dorsalmedial pre-motor region (Cz, FCz, C1) and the black diamonds represent the left temporal-parietal region (T7, TP7). **(B)** There was a significant positive correlation between ΔLMT and dwPLI between P4 and the left temporal-parietal region that survived correction for multiple comparisons. This association was such that stronger baseline connectivity indicated a rightward shift in attention following cTBS. **(C)** There was a significant positive correlation between ΔLMT and dwPLI between P4 and the dorsalmedial pre-motor region that did not survive correction for multiple comparisons. This association was such that stronger baseline connectivity indicated a rightward shift in attention following cTBS. *r*_*s*_, Spearman Rank correlation coefficient.

The strongest relationship was observed for a model of connectivity in the high beta band (fitted PLS model *R*^2^ = 0.51) which also had a strong predictive value (cross-validated *R*^2^ = 0.49, Table 1). This high beta model identified two clusters of electrodes that were approximately overlying a dorsalmedial pre-motor region (Cz, FCz, C1) and the left temporal-parietal region (T7, TP7) (see Figure 3). These clusters remained consistent across a wide range of thresholds used to generate the PLS model (0.23–0.80), suggesting this was a robust and consistent result. There were significant positive correlations between ΔLMT and dwPLI between the seed (P4) and each cluster (see Table 2). However, after applying Bonferroni correction for the 10 comparisons, only the correlation between ΔLMT and dwPLI between P4 and the cluster of electrodes approximating the left temporal-parietal region (T7, TP7) remained significant [*r*_*s*_ = 0.74, *p* = 0.04 (corrected)]. PLS models for theta, alpha, low beta and gamma frequencies identified electrode clusters that were not significantly associated with ΔLMT. The delta frequency PLS model did not identify any clusters of electrodes. Topographical plots for delta, theta, alpha, low beta and gamma are provided in the Supplementary Material.

**TABLE 1:** PLS models generated for ΔLMT following cTBS.

Frequency	Fitted R^2^	Cross validated R^2^	Number of electrode clusters
Delta	0.20	0.18	0
Theta	0.40	0.38	2
Alpha	0.55	0.25	3
Low Beta	0.40	0.25	2
High Beta	0.51	0.49	2
Gamma	0.39	0.26	1

**TABLE 2:** Correlation between ΔLMT and dwPLI values for connectivity between the seed (P4) and each cluster.

Frequency	Clusters	Result	Bonferroni corrected *p*
Theta	PO4 O2	*r*_*s*_ = 0.26, *p* = 0.39	*p* = 1.00
	C2, FC4, C6	*r*_*s*_ = 0.51, *p* = 0.08	*p* = 0.80
Alpha	F2, FC2, C2, CP2, CP4	*r*_*s*_ = 0.36, *p* = 0.23	*p* = 1.00
	PO4, PO6, PO8, O2	*r*_*s*_ = 0.50, *p* = 0.08	*p* = 0.80
	TP7, P7	*r*_*s*_ = 0.01, *p* = 0.98	*p* = 1.00
Low beta	PO6, PO8, P8	*r*_*s*_ = −0.31, *p* = 0.31	*p* = 1.00
	C3, CP3	*r*_*s*_ = −0.17, *p* = 0.58	*p* = 1.00
High beta	Cz, FCz, C1	*r*_*s*_ = 0.54, *p = 0.05	*p* = 0.50
	T7, TP7	*r*_*s*_ = 0.74, *p = 0.004	*p = 0.04
Gamma	O2, PO6, PO8	r = 0.15, *p* = 0.62	*p* = 1.00

*Significant (*p* < 0.05). r, Pearson’s correlation coefficient; *r*_*s*_, Spearman Rank correlation coefficient.

### Regression Model Combining Determinants of cTBS Response

A multiple regression combining the identified high beta functional connectivity network and recorded determinants of cTBS response was performed (*R*^2^ = 0.94, *p* = 0.01). The only significant independent variable was high beta dwPLI between P4 and the left temporal-parietal region (see Table 3).

**TABLE 3:** Multiple regression to predict ΔLMT with known determinants of cTBS response.

	Unstandardized beta estimate (SE)	Standardized beta	Confidence interval	*p*-value
Intercept	47.12 (20.61)		−5.85–100.91	0.071
Sex	−9.79 (5.81)	−0.27	−24.74–5.15	0.153
Age	−1.07 (0.66)	−0.24	−2.76–0.62	0.165
RMT	−0.08 (0.29)	−0.05	−0.82–0.67	0.801
IPAQ	0.00 (0.00)	−0.10	0.00–0.00	0.486
PSSQ	−0.03 (0.89)	−0.01	−2.31–2.25	0.974
High Beta dwPLI (P4–T7/TP7)	874.73 (221.98)	0.88	304.10–1,445.35	0.011*
High Beta dwPLI (P4–Cz/FCz/C1)	107.09 (183.68)	0.13	−365.08–579.26	0.585

Note that the only significant independent variable was High Beta dwPLI (P4–T7/TP7). Significance is indicated by *. dwPLI, debiased weighted phase lag index; IPAQ, international physical activity questionnaire; PSSQ, perceived stress scale questionnaire; RMT, resting motor threshold.

### EEG Connectivity Following cTBS Response

**FIGURE 4:**
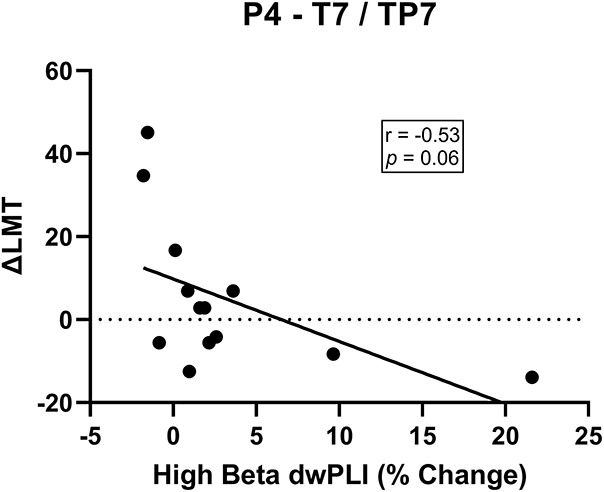
There was a non-significant trend for a negative association between change in high beta dwPLI between P4 and T7/TP7 electrode cluster and ΔLMT. Although not reaching statistical significance, this trend might indicate a decrease in high beta connectivity following cTBS is associated with a rightward shift in attention.

For connectivity between the seed (P4) and the region approximating the left temporal-parietal region (T7, TP7), there was no significance change in dwPLI at a group level from baseline to post-cTBS [*t*_(12)_ = 0.41, *p* = 0.69]. Investigation of responders [*t*_(6)_ = 0.90, *p* = 0.40] and non-responders [*t*_(5)_ = −1.38, *p* = 0.23] separately also revealed no significant change in connectivity of this network. There was a non-significant trend indicating that greater reduction in dwPLI following cTBS was correlated with a stronger rightward shift in attention (*r* = −0.53, *p* = 0.06; Figure 4). Analysis of the dorsalmedial pre-motor cluster (Cz, FCz, C1) did not reveal a change in dwPLI following cTBS or correlation with ΔLMT (all *p* > 0.11).

## Discussion

The ability to modulate brain activity with non-invasive stimulation provides a useful technique to probe human behavior or provide therapeutic treatments in disease. Despite these significant opportunities, there remain valid concerns regarding response variability. Here, we explored whether baseline functional connectivity with the right PPC predicted response to an inhibitory brain stimulation paradigm (cTBS) to induce change in spatial attention in healthy adults. To tease apart the specific role of baseline functional connectivity as a determinant of cTBS response, we controlled known factors such as age, gender, time of day, stress levels and physical activity that influence neuroplasticity induced with brain stimulation (Ridding and Ziemann, 2010). Our results suggest a model of baseline functional connectivity in the high beta band comprising a seed electrode overlying the right PPC and two clusters of electrodes approximating a dorsalmedial pre-motor region and the left temporal-parietal region was a strong determinant of behavioral change induced by cTBS. Further examination of each electrode cluster revealed stronger baseline connectivity between a network approximating the right PPC and the left temporal-parietal region was significantly correlated with a rightward shift in spatial bias following cTBS. Finally, in an exploratory investigation, we observed a trend suggesting that decrease in connectivity between the right PPC and the left temporal-parietal region following cTBS might be associated with a rightward shift in spatial bias, possibly suggesting this network has a role in behavioral change induced by cTBS to the PPC.

### Behavioral Response to PPC cTBS Is Variable

Several studies have investigated behavioral change induced by suppressive stimulation of the PPC. For example, cTBS to the right PPC has been shown to induce a rightward shift in visuospatial exploration behavior for up to 30 min post-stimulation (Nyffeler et al., 2008; Cazzoli et al., 2009a,b). Along similar lines, high frequency repetitive TMS to the left PPC appears to also induce a temporary rightward bias in spatial attention (Fierro et al., 2000). Despite evidence indicating it is possible to shift spatial attention and temporarily mimic neglect-like symptoms seen after right hemisphere stroke, it is evident that PPC stimulation can produce highly inconsistent responses, exhibited as variability in the direction and/or magnitude of behavioral change (Rizk et al., 2013; Chechlacz et al., 2015; Killington et al., 2016). Similar variability is observed when cTBS is applied to the right hemisphere as a therapeutic intervention in people with spatial neglect after stroke (Nyffeler et al., 2019). Our results confirm previous literature suggesting inter-individual variability in response to cTBS with approximately half of the participants demonstrating the expected behavioral response. Of note, these findings align well with the extensive literature around response variability following motor cortex cTBS (Hamada et al., 2013; Hinder et al., 2014; López-Alonso et al., 2014; Hordacre et al., 2015, 2017a).

### Functional Connectivity as a Determinant of cTBS Response

Our results suggest a role for functional connectivity as a determinant of cTBS applied to the right PPC to induce change in spatial attention. Specifically, stronger high beta functional connectivity between the stimulated right PPC, a dorsomedial pre-motor region and left temporal-parietal region was a marker of the anticipated rightward shift in spatial attention following cTBS. A separate analysis of each electrode cluster revealed that this outcome was primarily driven by connectivity between electrodes approximating the right PPC and the left temporal-parietal region. This network’s likely role as a marker of behavioral change is emphasized by the fact we controlled for other known determinants of brain stimulation, removing their possible influence on cTBS responses. This finding provides confidence that functional connectivity appears to be a new and independent determinant of cTBS response that might assist in improving response reliability by helping to decipher how theta burst stimulation interacts with the human cortex. Finally, we reported preliminary evidence that might suggest reduced connectivity of this network following cTBS is associated with rightward shift in spatial bias. Although we need to be cautious when interpreting this result as we did not include a sham cTBS condition, this novel finding provides some additional support for the importance of this high beta network. Future sham-controlled studies should explore the relationship between cTBS induced changes in physiology and behavior.

It could be that the high beta network we identified comprises regions belonging to the dorsal attention network (Corbetta and Shulman, 2011; Vossel et al., 2014). The dorsal attention network includes bilateral brain regions around the inferior parietal sulcus, frontal eye field and superior parietal lobule and previous literature provides evidence that it is possible to distinguish the dorsal attention network in the absence of a task (i.e., at rest) (Fox et al., 2006; He et al., 2007). This network is thought to support top-down voluntary allocation of attention by orientating to cues or stimuli (Corbetta and Shulman, 2011; Vossel et al., 2014). A recent study reported that right PPC brain stimulation increased resting state connectivity between the right PPC and left superior temporal gyrus, demonstrating ability of brain stimulation to modulate attentional networks (Schintu et al., 2021). Furthermore, previous studies have reported that structural integrity and microstructure of parieto-parietal white matter pathways contribute to behavioral response variability following cTBS to the PPC in both people with stroke and healthy adults (Chechlacz et al., 2015; Nyffeler et al., 2019; Schintu et al., 2021). It may be that individual differences in the functional network identified in this study might manifest through structural abnormalities in the interhemispheric white matter pathways. The role of functional networks in contributing to attentional deficits is demonstrated in people with stroke where there is evidence that spatial awareness depends not only on structural damage to white matter pathways, but are mediated by dysfunction in regions of the structurally intact dorsal attention network (Corbetta et al., 2005). Given these characteristics, we cautiously propose that the network identified here, with a seed overlying the right PPC and a cluster approximating the left temporal-parietal region might represent a portion of the dorsal attention network. How this network influences behavioral response to cTBS might be best explained by considering interhemispheric imbalance in neural activity. Specifically, cTBS to the right PPC, might act to induce imbalance in the bilateral dorsal network, leading to attentional deficits. However, it stands to reason if there is reduced network connectivity of the dorsal attention network at baseline, the interhemispheric imbalance effects of cTBS will be smaller.

Our finding that this network communicates *via* the high-beta band is supported by previous literature investigating attentional tasks. For example, in non-human primates, a visual search task that engaged top-down attentional processes was associated with stronger high beta activity across frontal and parietal dorsal regions (Buschman and Miller, 2007). Similarly, in humans, high beta oscillatory power correlated with frontoparietal connectivity and behavioral performance on a visual search and shooting task (Rogala et al., 2020). Furthermore, using TMS bursts to increase synchronization of high beta oscillations, several studies have demonstrated stronger long-range high beta synchronization between both the bilateral parietal cortices and a network including the right frontal eye field and right parietal region led to increased visual perception and detection (Quentin et al., 2015; Stengel et al., 2021).

### Future Direction

Functional connectivity may be worthy of consideration to improve the reliability of stimulation aftereffects. A next step to further disentangle the role of functional connectivity would be to explore causal relationships between beta connectivity and cTBS response [for example, see Hordacre et al. (2021a)]. Our preliminary findings suggest connectivity between the right PPC and left temporal-parietal region may be important. Furthermore, future studies should also explore whether this beta functional connectivity network has similar capacity to predict shift in spatial attention in people with spatial neglect following stroke. Such investigations might have the capacity to improve treatment efficacy for therapeutic applications of non-invasive brain stimulation. Some evidence to support this possibility comes from motor cortex direct current stimulation work where functional connectivity was identified as a predictor of physiological response (Hordacre et al., 2018). Understanding the mechanisms by which brain stimulation interact with the human cortex could prove useful for developing tailored brain stimulation models to guide therapeutic application in clinical populations (Hordacre et al., 2021b).

### Limitations

Results of this study should be considered with regards to several limitations. First, the sample size was relatively small and larger studies appear justified to further explore these findings. Second, individual magnetic resonance imaging was not available to enable neuronavigation to position the TMS coil. Instead we positioned the TMS coil at P4 based on the 10–20 EEG system which may not precisely represent the right PPC in each participant. However, a benefit of this approach is that the seed for functional connectivity analysis was precisely the cTBS application location. Finally, as with all scalp EEG recordings, it is difficult to know neural generators of recorded signals. To be conservative with our functional connectivity estimate, we used a metric known to bias against zero phase lag synchronization to avoid volume conduction and common reference problems. Furthermore, we cautiously referred to electrode clusters as approximately overlying cortical regions to avoid over interpretation of our findings.

## Conclusion

In summary, this study identified a determinant of cTBS response following stimulation of the right PPC. This network might represent components of the dorsal attention network. We demonstrated that stronger connectivity at baseline leads to a stronger response to cTBS, inducing a rightward bias shift when applied to the right PPC in healthy adults. These findings provide further support for the role of connectivity as a determinant of response to non-invasive brain stimulation. It may be that resting state connectivity could be used as a marker of responsiveness to theta burst stimulation applied to the PPC in future studies.

## Data Availability Statement

The original contributions presented in the study are included in the article/Supplementary Material, further inquiries can be directed to the corresponding author/s.

## Ethics Statement

This study was reviewed and approved by the University of South Australia Human Research Ethics Committee. All participants provided their written informed consent prior to participating in this study.

## Author Contributions

JM was responsible for drafting the manuscript. TL and BH reviewed draft iterations. All authors contributed to study design, data analysis, and interpretation.

## Conflict of Interest

The authors declare that the research was conducted in the absence of any commercial or financial relationships that could be construed as a potential conflict of interest.

## Publisher’s Note

All claims expressed in this article are solely those of the authors and do not necessarily represent those of their affiliated organizations, or those of the publisher, the editors and the reviewers. Any product that may be evaluated in this article, or claim that may be made by its manufacturer, is not guaranteed or endorsed by the publisher.
