# Supplementary material for: Parietal Cortex Connectivity as a Marker of Shift in Spatial Attention Following Continuous Theta Burst Stimulation
Source: Front Hum Neurosci. 2021 Sep 8;15:718662. doi: 10.3389/fnhum.2021.718662 (PMC8455944; doi:10.3389/fnhum.2021.718662)
Supplement: Supplementary file 1 [file Data_Sheet_1.docx]

Supplementary Material

| **ID** | **Age (years)** | **Sex (F/M)** | **PSSQ** | **IPAQ (MET)** | Accuracy Pre-intervention Session | Accuracy Post-intervention Session |
| --- | --- | --- | --- | --- | --- | --- |
| 1 | 22 | F | Mod (17/40) | High (2,293) | 75.0 | 77.8 |
| 2 | 18 | F | Low (12/40) | High (3,862) | 68.1 | 66.0 |
| 3 | 21 | M | Mod (20/40) | High (4,540) | 63.9 | 79.9 |
| 4 | 21 | M | Mod (20/40) | High (13,333) | 80.6 | 77.8 |
| 5 | 28 | M | Mod (19/40) | High (2,388) | 75.7 | 81.3 |
| 6 | 28 | F | Mod (21/40) | High (8,678) | 68.1 | 63.2 |
| 7 | 21 | F | Low (12/40) | High (6,732) | 63.2 | 76.4 |
| 8 | 27 | F | Low (10/40) | Mod (858) | 50.0 | 58.3 |
| *9 | 28 | M | Mod (20/40) | High (22,450) | 16.0 | 77.8 |
| 10 | 30 | F | Mod (16/40) | High (6,768) | 60.4 | 49.3 |
| 11 | 27 | F | Mod (18/40) | Mod (1307) | 64.6 | 68.8 |
| 12 | 28 | M | Mod (19/40) | High (3,969) | 59.7 | 66.0 |
| 13 | 22 | F | Mod (21/40) | High (1,540) | 68.8 | 81.3 |
| 14 | 29 | F | Low (13/40) | Mod (1,232) | 70.8 | 69.4 |
|  | Range:18-30 | Ratio: 9 F: 4 M | Range:10-21 | Range:858-13333 | **63.2** | **70.9** |
| **Mean/(SD)** | 24.8 (4.0) |  | 16.8 (3.8) | 4423.1(3633.7) |  |  |

**TABLE 1: Individual Participant Characteristics**

Abbreviations: **PSS**: Perceived Stress Scale**, IPAQ**: International Physical Activity Questionnaire, **MET**: A multiple of your estimated resting energy expenditure, represents the amount of energy expended carrying out physical activity (minutes in one week), **SD**: Standard Deviation, *: Participant excluded from study.


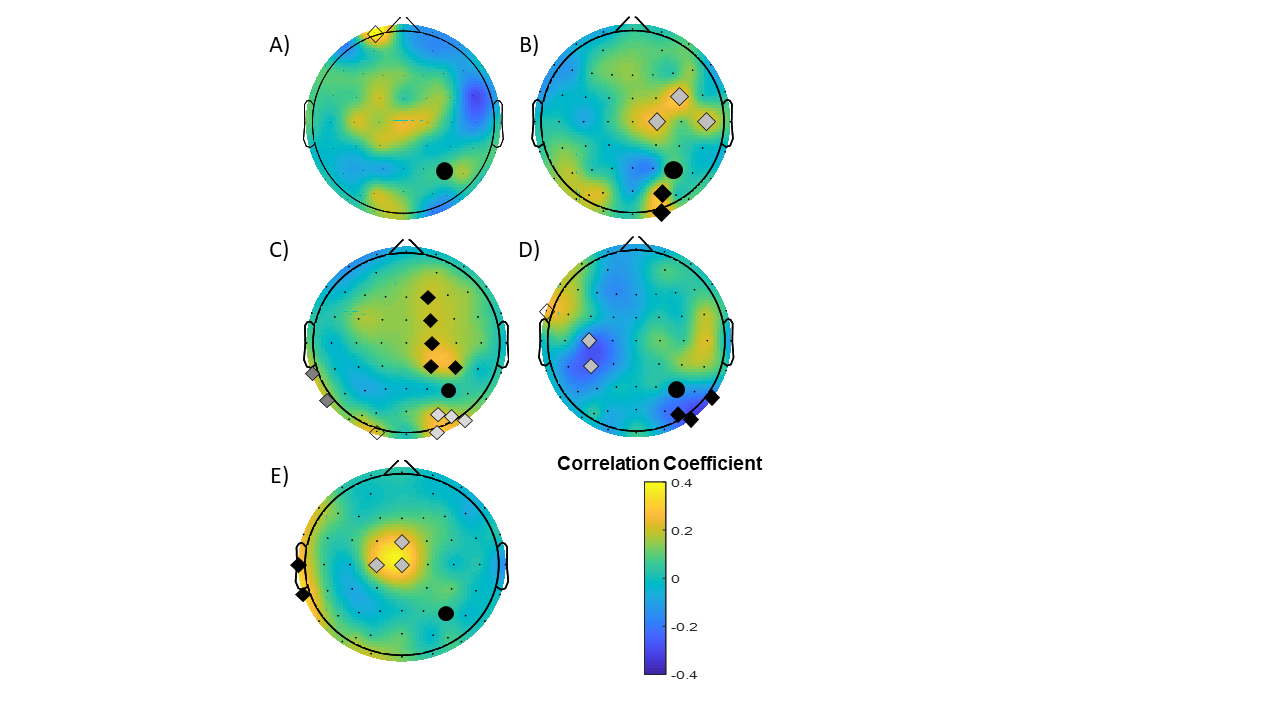


**Figure 1: Topographic plots for PLS analysis of connectivity and ΔLMT.** Figures are presented in delta (A), theta (B), alpha (C), low beta (D) and gamma (E) bands. The seed electrode is shown in a black filled circle. Electrode clusters are shown in filled diamonds (different colours for each separate cluster). Electrodes not in a cluster are shown in non-filled diamonds.
